# Association between nonstandard work schedules and employees’ self-rated mental health with perceived work stress, work–family conflict and job satisfaction as psychosocial mediators: a national study in China

**DOI:** 10.3389/fpsyg.2025.1561653

**Published:** 2025-05-29

**Authors:** Zhan Wang, Guangsheng Zhang

**Affiliations:** ^1^The College of Economics and Management, Shenyang Agricultural University, Shenyang, China; ^2^Business School, Shenyang University, Shenyang, China; ^3^Business School, Liaoning University, Shenyang, China

**Keywords:** nonstandard work schedules, mental health, work stress, work–family conflict, job satisfaction, shift work, job demands-resources model

## Abstract

**Background:**

Nonstandard work schedules are prevalent across the industrialized world. While prior research indicates nonstandard work schedules lead to poor mental health, little research has explored the psychosocial pathways underlying the association between work schedule and mental health. This study aimed to fill this gap by testing for the mediating roles of perceived work stress, perceived work–family conflict and perceived job satisfaction in the association between nonstandard work schedules and employees’ self-rated mental health.

**Methods:**

Using a nationally representative sample of data from the Chinese General Social Survey (CGSS) in 2021 (*N* = 1857), and using the Process v4.1 for SPSS, we examined the association between nonstandard work schedules and employees’ self-rated mental health and estimated the independent and joint mediation effects of perceived work stress, perceived work–family conflict and perceived job satisfaction.

**Results:**

A total of 1857 employees participated in the final analysis. Of these, 1,331 employees (71.7%) work fixed day shifts, 24 employees (1.3%) work fixed night shifts, 206 employees (11.1%) work rotating shifts, 243 employees (13.1%) work irregular schedules and 53 employees (2.8%) work other schedules. Nonstandard work schedule was negatively correlated with employees’ self-rated mental health and perceived job satisfaction, and positively correlated with perceived work stress and work–family conflict (*p* < 0.001). The independent mediation effects of perceived work stress, perceived work–family conflict and perceived job satisfaction was 17.3, 22.4 and 16.5%, respectively. The joint effect of all three mediators mediated about 36.2% of the relationship between nonstandard work schedules and employees’ self-rated mental health. Sensitivity analyses revealed that rotating shift (*p* < 0.05) and irregular schedule (*p* < 0.001) were negatively associated with employees’ self-rated mental health, perceived job satisfaction fully mediated the association between rotating shift and employees’ self-rated mental health, while perceived work stress, work–family conflict and job satisfaction jointly and partially mediated the association between irregular schedule and employees’ self-rated mental health (the joint effect of all three mediators mediated about 42.4% of the relationship).

**Conclusion:**

Perceived work stress, perceived work–family conflict and perceived job satisfaction mediated the relationship between nonstandard work schedules and employees’ self-rated mental health. The findings advance understanding of the psychosocial mechanisms underlying the association between work schedule and mental health.

## 1 Introduction

Countries around the world have shifted to a 24/7 economy (an economy that operates 24 h per day and 7 days per week), which means increasing numbers of employees are working nonstandard hours (Smith et al., 2023). Nonstandard work schedules are generally defined in the literature as work that occurs outside of the traditional daytime and weekly Monday to Friday 9 am–5 pm schedule (Smith et al., 2023; Taiji and Mills, 2020; Totterdell, 2005). Nonstandard work schedules are known to affect mental health, not only through biological pathway, in which the body’s circadian rhythms are affected and thus leading to physiological disturbances and the inability to cope, but also through social pathway (Haines et al., 2008). However, current research and evidence on the social or psychosocial pathway by which nonstandard work schedules may lead to poor mental health is limited. To our best knowledge, aside from three studies to date have attempted to assess the mediating role of work–family conflict in the association between nonstandard work schedules and mental health, which were conducted in Australia (Zhao et al., 2021a), Canada (Haines et al., 2008) and the USA (Cho, 2018), no study has assessed other social or psychosocial mediators in the association between nonstandard work schedules and mental health. Given to Totterdell’s (2005) proposal that more research is needed to elucidate the precise biological, psychological, and social pathways, we believe that more research is needed to examine the potential psychosocial mechanisms underlying the association between nonstandard work schedules and mental health.

In this study, using data from the nationally representative Chinese General Social Survey, we attempted to address the identified gaps by assessing the mediating roles of perceived work stress, perceived work–family conflict and perceived job satisfaction in the association between nonstandard work schedules and employees’ self-rated mental health.

### 1.1 Contributions of the research

Our study contributes to the literature by demonstrating the psychosocial mechanisms of perceived work stress, perceived work–family conflict and perceived job satisfaction in the study of association between work schedule and mental health, since little research has explored the psychosocial pathways underlying the association between work schedule and mental health. Our study is the first, to our knowledge, to explicitly test and confirm the mediating roles of perceived work stress and job satisfaction in the association between work schedule and mental health. This study also used a unique data set from a nationally representative survey (Wang et al., 2024) which the latest 2021 wave adds the quality of work topical module. To our knowledge, this is the first nationally representative study in China to investigate work schedules.

## 2 Background and theoretical frameworks

### 2.1 Importance of understanding employees’ mental health

Employee mental health is a central issue in today’s global workplace (Kim K. Y. et al., 2023; Kim S. E. et al., 2023). Poor mental health can affect employees’ work efficiency and job performance (Chen et al., 2022). Depressive symptoms are frequent and cause considerable suffering for the employees as well as financial loss for the employers (Theorell et al., 2015). Furthermore, mental health problems have been acknowledged as a major public health issue (Zhao et al., 2019). For example, depression accounts for 4.3% of the global burden of disease and incidence, with mental disorders worldwide predicted to cost US $16.3 million by 2030 (Torquati et al., 2019). This suggests that understanding the social determinants of employees’ mental health is important.

### 2.2 Prevalence of nonstandard work schedules

Nonstandard work schedules have long existed (Bolino et al., 2021). Because of the demand for round-the-clock service from various vital sectors such as health, security, transport and communication, the technical need for maintaining continuous process industries, and the development in industry and social sector, nonstandard work schedules become more and more inevitable (Vogel et al., 2012). Nonstandard work schedules are prevalent across the industrialized world (Kim et al., 2025; Wang, 2023), although their prevalence is difficult to compare due to the use of different samples and definitions (Li et al., 2014). In the UK, data from the UK Household Longitudinal Study showed that 28.2% of employees worked nonstandard hours (Weston et al., 2024). In Sweden, data from the Swedish Longitudinal Occupational Survey of Health showed that 19.72% of Swedish workers have a shift work with nights, 24.49% of Swedish workers have a shift work without nights, and only 55.78% of Swedish workers have a daywork (Tucker et al., 2021). In South Korea, data from the Korea Labor and Income Panel Survey showed that 10.4% of workers worked shift work (Jung et al., 2022).

### 2.3 Work schedule and mental health

A number of studies have provided evidence of an association between nonstandard work schedules and mental health (Brown et al., 2020; Han, 2023; Harris et al., 2024; Lee et al., 2016; Niedhammer et al., 2022). Nonstandard work schedules can disrupt circadian rhythms and contribute to sleep disturbances (Coelho et al., 2023; Frazier, 2023; Jeon and Kim, 2022; Kim K. Y. et al., 2023; Kim S. E. et al., 2023; Sateia, 2014; Weng and Chang, 2024; Weston et al., 2024; Zhang et al., 2024). Consequently, sleep disturbances frequently underlie the mental health consequences of nonstandard work schedules, and mediated the association between nonstandard work schedules and mental health (Brown et al., 2020; Cheng et al., 2023; Das and Palo, 2024; Frazier, 2023; McNamara and Robbins, 2023; Zhang et al., 2022).

Nonstandard work schedules may negatively associate with mental health through channels other than sleep as well (Chen et al., 2025; Sierpińska and Ptasińska, 2023). However, besides sleep disturbances, much less is known about what is a clear mechanism underlying the association between nonstandard work schedules and mental health. In the following sections, we propose and outline the theoretical underpinnings linking nonstandard work schedules to mental health with the focus on the psychosocial pathways of perceived work stress, work–family conflict and job satisfaction.

### 2.4 The mediating role of work stress

Theoretically, the job demands-resources (JD-R) model is one of the most popular and influential models of work stress in the literature (Roskams et al., 2021), which provides a theoretical framework linking nonstandard work schedules, stress and mental health. The model clearly expands earlier models of work-related stress and burnout, such as Karasek’s (1979) job demands-control model (which predicts and explains that job strain, occurs when job demands are high and job decision latitude is low, is related to symptoms of mental strain) (Demerouti et al., 2001; Karasek, 1979). Job demands refer to those physical, social, or organizational aspects of the job that require sustained physical or mental effort and are therefore associated with certain physiological and psychological costs (e.g., exhaustion), and nonstandard work schedule is clearly identified as a typical example of job demands (Demerouti et al., 2001; Roskams et al., 2021). The demanding aspects of work (e.g., nonstandard work schedules) lead to exhaustion (Hong, 2025; Ong and Johnson, 2023), which is a state that closely resembles traditional stress reactions studied in occupational stress research, conceptually (Demerouti et al., 2001). Further, a lack of job resources leads to a state of disengagement, and the combination of exhaustion and disengagement is symptomatic of burnout (Romo et al., 2025; Roskams et al., 2021), which is in turn associated with various negative outcomes such as impaired mental health (Chen et al., 2025; Fix and Powell, 2024; Gawlik et al., 2025; Kerr et al., 2025; Koutsimani et al., 2019).

Unsurprisingly, a number of quantitative studies have found that working nonstandard hours can lead to higher levels of work stress (Bolino et al., 2021; Castillo et al., 2020; Golden, 2015; Son and Lee, 2021; Yuan and Fang, 2024). From a functional neuroimaging perspective, working on long-term shift had decreased brain functional activity and connectivity in the right frontoparietal network, which were associated with burnout, stress, and depression conditions (Dong et al., 2024).

In turn, a robust body of literature has indicated that perceived work-rated stress is a strong predictor of mental health (Chireh et al., 2023; Du et al., 2023; Niedhammer et al., 2021; Theorell et al., 2015). One possible channel through which work stress affect mental health is that work stress can affect sleep quality (Xu et al., 2024; Matti et al., 2024), although Son and Lee (2021) found that high work stress had stronger associations with suicidal ideation than poor sleep quality in shift workers.

Given the Job Demands-Resources model, and given that nonstandard work schedules can lead to higher levels of work stress and the clear indication that perceived work stress is a risk factor for mental health, we anticipate that perceived work stress will mediate the association between nonstandard work schedules and employees’ self-rated mental health.

### 2.5 The mediating role of work–family conflict

Theoretically, the work–family conflict model provides a useful theoretical framework for understanding the mechanism through which nonstandard work schedules affect workers’ mental health (Cho, 2018; Haines et al., 2008; Zhao et al., 2021a). The work–family conflict model proposed that work–family conflict is a form of inter-role conflict in which the role pressures from the work and family domains are mutually incompatible in some respect, and any role characteristic that affects a person’s time involvement, strain, or behavior within a role can produce conflict between that role and another role (Greenhaus and Beutell, 1985). Shift work may simultaneously produce both time-based and strain-based work–family conflict (Cho, 2018; Haines et al., 2008; Wöhrmann et al., 2020; Zhao et al., 2021a). Nonstandard work schedules require employees to work at times that are most socially valuable, such as evenings and weekends, during which most family and other social activities take place, that results in time-based work–family conflict (Wöhrmann et al., 2020). Working nonstandard hours often also leads to sleep deprivation and fatigue, which, in turn, leads to irritability and strain in family interactions, that results in strain-based work–family conflict (Zhao et al., 2021a).

Empirical studies have found the association between nonstandard work schedules and work–family conflict (Al-Hammouri and Rababah, 2023; Ambiel et al., 2024; Lambert et al., 2023; Tammelin et al., 2017; Zhao et al., 2021b), and the strength of this association varies significantly between countries (Taiji and Mills, 2020).

In addition, work and family roles and the balance between the two are important for workers’ mental health (Wang et al., 2007), and the association between work–family conflict and mental health was moderated by socioeconomic status, family structure, job conditions, educational mismatch, resilience and social support (Huang et al., 2024; Lee et al., 2022; Song et al., 2024; Zhang et al., 2020). Given these considerations, we anticipate that working nonstandard schedules will lead to greater perceived work–family conflict, which in turn will augment the risk of experiencing poor mental health.

### 2.6 The mediating role of job satisfaction

Theoretically, there is an inherent factor causing shift workers to be less satisfied with their jobs than day workers, and the dissatisfaction may be created by the shift workers’ non-congruent role in society in relationship to the time schedule of the majority of the community (Dunham, 1977). Specifically, Dunham’s (1977) theoretical analysis is that “there appears to be a 24-h cyclical rhythm of not only bodily functions but social and personal functions structured by community practices. The shift worker is often out of phase with the rest of the community in this cycle and becomes the deviant within the society. This deviancy ‘costs’ the shift worker through adjustment problems with important non-physiological functions.”

The literature has suggested that nonstandard work schedules may lead to feelings of job dissatisfaction. Among nurses and physicians, work schedule conflicts were one of the least satisfying aspects of work (House et al., 2022), and comparing to day shift nurses, irregular shift nurses (Bagheri Hosseinabadi et al., 2019), fixed night shift nurses and rotating shift nurses (Kim et al., 2024) suffered from lower job satisfaction. In addition, crew scheduling is one of the antecedents of employee dissatisfaction in airlines (Han et al., 2024).

In turn, a large body of quantitative research has indicated that job satisfaction contributes to mental health (Chireh et al., 2023; Chong et al., 2023; Jilili et al., 2023; Monahan et al., 2023; Wang and Derakhshan, 2025; Worley et al., 2023; Yang et al., 2024; Yun et al., 2024; Zhou et al., 2023), coving a variety of occupations in different countries.

Given Dunham’s (1977) theory, and given that nonstandard work schedules may lead to feelings of job dissatisfaction and the clear indication that job satisfaction contributes to mental health, we anticipate that perceived job satisfaction will mediate the association between nonstandard work schedules and employees’ self-rated mental health.

## 3 Present study

In summary, theories and previous research findings provide grounds for anticipating that nonstandard work schedules may affect mental health, and perceived work stress, perceived work–family conflict and perceived job satisfaction may account for the association between nonstandard work schedules and mental health. However, little research has explicitly test and confirm the psychosocial pathways underlying the association between work schedule and mental health. To examine the potential psychosocial mechanisms in the association between nonstandard work schedules and mental health, based upon prior research and theoretical analysis, the present study tests the following hypotheses among a nationally representative sample from China:

*H1*: Nonstandard work schedules will be negatively associated with employees’ self-rated mental health.

*H2*: The association between nonstandard work schedules and employees’ self-rated mental health will be mediated by perceived work stress.

*H3*: The association between nonstandard work schedules and employees’ self-rated mental health will be mediated by perceived work-family conflict.

*H4*: The association between nonstandard work schedules and employees’ self-rated mental health will be mediated by perceived job satisfaction.

## 4 Methods

### 4.1 Data

We use the latest data of Chinese General Social Survey (CGSS) in 2021. The CGSS is the earliest national representative continuous survey project run by academic institution in China mainland. It collects quantitative data about measures of social structure, quality of life, and underlying mechanisms linking social structure and quality of life, to systematically monitor the changing relationship between social structure and quality of life in China (Bian and Li, 2012). CGSS was launched in 2003 and had included 12 waves so far: 2003, 2005, 2006, 2008, 2010, 2011, 2012, 2013, 2015, 2017, 2018 and 2021. Latest 2021 wave added quality of work topical module, which exactly fulfill our need for research.

This study used the latest wave of CGSS, which surveyed residents in 19 provinces of China. The original sample size was 8,148, of which 1,978 were employees. After excluding observations with missing data on at least one of the study variables in addition to income, the final sample was restricted to 1,857 observations. Income as a control variable has 108 missing values still and we impute them using univariate imputation (Royston, 2004) through other control variables in order to reduce the loss of sample size. Among the 1,857 observations, males accounted for 53.15% and females accounted for 46.85%. In terms of age, 19.55% were between 18 and 29 years old, 28.76% were between 30 and 39 years old, 25.15% were between 40 and 49 years old, 20.19% were between 50 and 59 years old, and 6.35% were 60 years old or older. In terms of education level, 11.58% had primary school education or below, 25.47% had junior high school education, 20.62% had high school education, 21.16% had a junior college degree, 17.82% had a bachelor’s degree, and 3.34% had a graduate degree. In addition, in terms of work schedule, 71.67% had a fixed day shift, 1.29% had a fixed night shift, 11.09% had a rotating shift, 13.09% had an irregular schedule, and 2.85% had other schedules.

### 4.2 Measures

In this study, the outcome variable is self-rated mental health, the explanatory variable is nonstandard work schedules, the mediating variables are perceived work stress, perceived work–family conflict and perceived job satisfaction, the control variables are self-rated physical health, overtime work, changing jobs during COVID-19 and demographic characteristics (include gender, age, education, income, political status, marital status, family economic status and regions).

#### 4.2.1 Self-rated mental health

Consistent with previous research (Cho, 2018), self-rated mental health is measured by asking the question “During the past 4 weeks, how often you felt depressed?” Response options included “always,” “often,” “sometimes,” “rarely” and “never.” Responses were coded from 1 = “always” to 5 = “never.” Higher numbers indicate greater levels of mental health.

#### 4.2.2 Nonstandard work schedules

2021 CGSS quality of work module asks the question, “Which of the following best describes your usual work schedule?” Response options included “fixed day shift,” “fixed night shift,” “rotating shift,” “irregular schedule” and “other work schedules.” Consistent with previous research (Castillo et al., 2020; Cho, 2018; Haines et al., 2008; Han, 2023; Kachi et al., 2021; Lee et al., 2016; Tammelin et al., 2017; Weston et al., 2024; Zhao et al., 2021b), fixed day shift was categorized as “standard work schedules” and was coded as 0, while other four options were categorized as “nonstandard work schedules” and were coded as 1. In addition to this broad binary measure, each response option was a category of work schedules in sensitivity analysis.

#### 4.2.3 Perceived work stress

Perceived work stress is measured by asking the question “How often do you find your work stressful?” Response options included 1 = “always,” 2 = “often,” 3 = “sometimes” and 4 = “never.” Responses were reverse coded in this study so that higher values indicate more perceived work stress.

#### 4.2.4 Perceived work–family conflict

Consistent with previous research (Cho, 2018), perceived work–family conflict is measured by asking the question “In general, how often you felt that your job interfere with your family life?” Response options included 1 = “always,” 2 = “often,” 3 = “sometimes,” 4 = “rarely” and 5 = “never.” Responses were reverse coded in this study so that higher numbers indicate more perceived work–family conflict.

#### 4.2.5 Perceived job satisfaction

Perceived job satisfaction is measured by asking the question “In general, how satisfied would you say you are with your job?” Response options included 1 = “very satisfied,” 2 = “moderately satisfied,” 3 = “neither satisfied nor dissatisfied,” 4 = “a little dissatisfied” and 5 = “very dissatisfied.” Responses were reverse coded in this study so that higher numbers indicate greater levels of perceived job satisfaction.

#### 4.2.6 Control variables

Self-rated physical health is measured by asking the question “In general, how would you rate your physical health?” Response options included “Poor,” “Fair,” “Good,” “Very good” and “Excellent.” Responses were coded from 1 = “poor” to 5 = “excellent.” Higher numbers indicate greater levels of physical health.

Overtime work is measured by asking the question “Did you work overtime last week?” Respondents who responded “no” were coded as 0 and respondents who responded “yes” were coded as 1.

Changing jobs during COVID-19 is measured by asking the question “Compared to before the COVID-19, which of the following descriptions best fits your current situation?” Response options included “I did not change my job,” “I lost my job due to COVID-19, but I have a new job now,” “I did not have a job before COVID-19, but now I have a job” and “I changed my job, but not because of COVID-19.” The first option was categorized as “did not change jobs during COVID-19” coded 0, and other options were categorized as “change jobs during COVID-19” coded 1.

Demographic characteristics include gender (0 = males, 1 = females), age (equals “2021 - birth year”), education (1 = primary school or below, 2 = junior high school, 3 = high school, 4 = associate/junior college, 5 = bachelor’s, 6 = graduate), income (equals “ln (1 + personal annual income),” and was winsorized at 1 and 99% levels), political status (1 = Communist Party members), marital status (1 = currently married), family economic status (1 = far below average, 2 = below average, 3 = average, 4 = above average, 5 = far above average, compared with local families in general) and regions. Eastern region (reference) equals one if respondents’ residence is Beijing, Hebei, Jiangsu, Zhejiang, Fujian, Shandong or Liaoning, and zero otherwise. Central region equals one if respondents’ residence is Shanxi, Anhui, Jiangxi, Henan, Hubei or Hunan, and zero otherwise. Western region equals one if respondents’ residence is Inner Mongolia, Guangxi, Chongqing, Shaanxi, Gansu or Ningxia, and zero otherwise.

### 4.3 Analytical strategy

SPSS 24.0 was used in this study. First, we conducted descriptive analysis for each of the study variables to understand the overall sample. Second, we computed the correlation matrix of our dependent variable, main independent variable, and mediators. Third, we conducted common method bias test to assess the potential for common method variance. Forth, we examined the association between nonstandard work schedules and self-rated mental health and estimated the independent and joint mediation effects of perceived work stress, work–family conflict and job satisfaction, using the Process v4.1 for SPSS (Hayes, 2017; Preacher and Hayes, 2008). We used 10,000 bootstrapped resamples to describe the 95% confidence intervals for effect that are statistically significant if their confidence intervals do not contain zero. Fifth, a sensitivity analysis was conducted to divide and compare each group of work schedules.

## 5 Results

### 5.1 Descriptive statistics

Table 1 presents the descriptive statistics for all study variables. The mean values of self-rated mental health, perceived work stress, work–family conflict and job satisfaction were 4.13 (SD = 0.93), 1.95 (SD = 0.94), 1.98 (SD = 1.05), and 3.67 (SD = 0.84), respectively. The mean value of self-rated mental health among standard work schedules and non-standard work schedules is 4.18 and 4.00, respectively (effect size of Cohen’s d = 0.20, *p* < 0.001). The mean value of perceived work stress among standard work schedules and non-standard work schedules is 1.90 and 2.07, respectively (effect size of Cohen’s d = 0.18, *p* < 0.001). The mean value of perceived work–family conflict among standard work schedules and non-standard work schedules is 1.90 and 2.17, respectively (effect size of Cohen’s d = 0.26, *p* < 0.001). The mean value of perceived job satisfaction among standard work schedules and non-standard work schedules is 3.73 and 3.50, respectively (effect size of Cohen’s d = 0.29, *p* < 0.001). *T*-tests show a lower level of self-rated mental health and perceived job satisfaction, and a higher level of perceived work stress and work–family conflict in non-standard work schedule group, compared with the standard work schedule group.

**Table 1:** Descriptive statistics (*N* = 1,857).

Variable	*M* or %	SD	Range
Self-rated mental health	4.13	0.93	1–5
Work schedule			
Standard work schedules (reference)	71.67%		0 = no, 1 = yes
Non-standard work schedules	28.33%		0 = no, 1 = yes
Fixed night shift	1.29%		0 = no, 1 = yes
Rotating shift	11.09%		0 = no, 1 = yes
Irregular schedule	13.09%		0 = no, 1 = yes
Other schedules	2.85%		0 = no, 1 = yes
Perceived work stress	1.95	0.94	1–4
Perceived work–family conflict	1.98	1.05	1–5
Perceived job satisfaction	3.67	0.84	1–5
Self-rated physical health	3.87	0.87	1–5
Overtime work	35.22%		0 = no, 1 = yes
Changing jobs during COVID-19	11.74%		0 = no, 1 = yes
Female	46.85%		0 = no, 1 = yes
Age	41.25	12.15	18–77
Education	3.18	1.38	1–6
Income	10.48	2.08	0–13.12
Communist Party members	16.69%		0 = no, 1 = yes
Married	71.73%		0 = no, 1 = yes
Family economic status	2.71	0.72	1–5
Regions			
Eastern region (reference)	54.07%		0 = no, 1 = yes
Central region	29.29%		0 = no, 1 = yes
Western region	16.64%		0 = no, 1 = yes

### 5.2 Correlation analysis

Correlation matrices for dependent, main independent and mediator variables are reported in Table 2. As shown in Table 2, the correlations between self-rated mental health, non-standard work schedule, perceived work stress, perceived work–family conflict and perceived job satisfaction are all significant at the *p* < 0.001 level.

**Table 2:** Correlation matrix for dependent, main independent, and mediator variables.

Variables	1	2	3	4	5
1 Self-rated mental health	1				
2 Non-standard work schedules	−0.09***	1			
3 Perceived work stress	−0.23***	0.08***	1		
4 Perceived work–family conflict	−0.20***	0.12***	0.39***	1	
5 Perceived job satisfaction	0.22***	−0.13***	−0.25***	−0.31***	1

***p < 0.001.

### 5.3 Common method bias test

Harman’s single-factor test was conducted to assess the potential for common method variance. The results showed that the variance explained by the first factor was 32.192%, which was below the acceptable threshold of 40%. Therefore, this suggests that significant common method bias was not present in this study.

### 5.4 Mediating effect analysis

**Figure 1:**
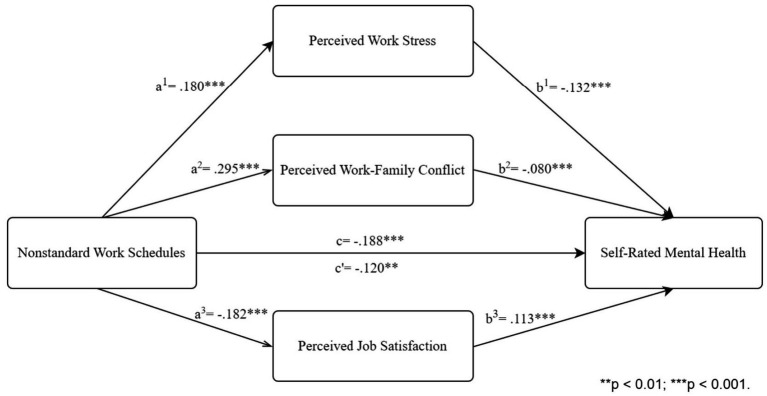
Results of the simultaneous mediation model.

To examine the association between nonstandard work schedules and employees’ self-rated mental health and estimate the independent and joint mediation effects of perceived work stress, work–family conflict and job satisfaction, we applied Hayes’s (2017) SPSS PROCESS v4.1 (Component Model 4), with 10,000 replicates and 95% CI. The results are shown in Table 3. First, nonstandard work schedules could significantly and negatively predict mental health (*β* = −0.188, *p* < 0.001), the total effect of nonstandard work schedules on self-rated mental health is −0.188 (Bootstrap 95% CI: −0.277, −0.099), and the direct effect of nonstandard work schedules on self-rated mental health is −0.120 (Bootstrap 95% CI: −0.208, −0.033), accounting for 63.8% of the total effect. Second, the independent mediation analysis showed that perceived work stress (accounting for 17.3% of the total effect), perceived work–family conflict (22.4%), and perceived job satisfaction (16.5%) significantly mediated the association between nonstandard work schedules and employees’ self-rated mental health. Third, the joint effect of perceived work stress, work–family conflict and job satisfaction mediated 36.2% of the total effect of nonstandard work schedules on employees’ self-rated mental health (Figure 1).

**Table 3:** Mediation of association between work schedule and mental health (*N* = 1,857).

Variables	Effect	Bootstrapped SE	Boot 95% CI	PME
Total effect	−0.188***	0.045	[−0.277, −0.099]	
Perceived Work stress	−0.033**	0.010	[−0.054, −0.014]	0.173
Perceived Work–family conflict	−0.042***	0.011	[−0.065, −0.024]	0.224
Perceived Job satisfaction	−0.031**	0.010	[−0.052, −0.014]	0.165
Joint indirect effect	−0.068***	0.015	[−0.099, −0.041]	0.362

SE, standard error; CI, confidence interval; PME, proportion of the effect mediated. **p < 0.01, ***p < 0.001.

### 5.5 Sensitivity analysis

Although many of previous studies lumped ‘any arrangement of daily working hours that differs from the standard daylight hours’ together labeled “nonstandard work schedule” or “shift work” and did not divide different types of nonstandard work schedules in their analysis given the small share of participants engaged in each of the detailed nonstandard work schedules (Castillo et al., 2020; Haines et al., 2008; Han, 2023; Jung et al., 2022; Lee et al., 2016; Tammelin et al., 2017; Weston et al., 2024), we are aware that dividing and comparing each group of work schedules would strengthen our findings. The mean values of self-rated mental health among fixed day shift, fixed night shift, rotating shift, irregular schedule and other schedules are 4.18, 4.17, 4.03, 3.95 and 4.00, respectively, but the differences of the mean values are significant only within two pairs (fixed day shift vs. rotating shift; fixed day shift vs. irregular schedule) due to the small sample size of “fixed night shift” (*n* = 24) and “other schedules” (*n* = 53). As shown in Table 4, controlling for the studied sociodemographic variables, rotating shift was significantly and negatively associated with employees’ self-rated mental health (β = −0.132, *p* < 0.05) and perceived job satisfaction (β = −0.156, *p* < 0.01), irregular schedule was significantly and negatively associated with employees’ self-rated mental health (β = −0.257, *p* < 0.001) and perceived job satisfaction (β = −0.251, *p* < 0.001), and significantly and positively associated with perceived work stress (β = 0.328, *p* < 0.001) and perceived work–family conflict (β = 0.473, *p* < 0.001). Furthermore, perceived job satisfaction fully mediated the association between rotating shift and employees’ self-rated mental health, and perceived work stress, perceived work–family conflict and perceived job satisfaction independently and jointly mediated the association between irregular schedule and employees’ self-rated mental health (the proportion of the joint mediation effects is 42.4%).

**Table 4:** Sensitivity analysis: regression coefficients of regression models (*N* = 1,857).

Variables	Self-rated mental health	Perceived work stress	Perceived work–family conflict	Perceived job satisfaction	Self-rated mental health
Fixed night shift	0.017	0.063	0.169	0.147	0.022
Rotating shift	−0.132*	0.056	0.136	−0.156**	−0.097
Irregular schedule	−0.257***	0.328***	0.473***	−0.251***	−0.148*
Other schedules	−0.185	0.051	0.172	−0.116	−0.151
Perceived work stress					−0.132***
Perceived work–family conflict					−0.080***
Perceived job satisfaction					0.112***
Self-rated physical health	0.365***	−0.090***	−0.090**	0.116***	0.333***
Overtime work	−0.119**	0.290***	0.476***	−0.226***	−0.017
Changing jobs	−0.204**	0.046	−0.081	−0.007	−0.204**
Female	−0.105*	−0.090*	−0.080	0.083*	−0.133**
Age	0.005*	−0.008**	−0.015***	0.014***	0.001
Education	−0.041*	0.077***	0.067**	0.037*	−0.030
Income	−0.020*	0.040***	0.054***	−0.013	−0.009
Communist party members	0.123*	−0.024	0.0429	0.065	0.116*
Married	0.055	0.136**	0.102	−0.002	0.081
Family economic status	0.079**	−0.065*	−0.079*	0.235***	0.037
Central region	−0.035	0.045	−0.001	−0.032	−0.025
Western region	−0.127*	0.160**	0.048	−0.078	−0.093

*p < 0.05; **p < 0.01; ***p < 0.001.

## 6 Discussion

The aim of our study was to extend the line of the work schedule—mental health ideation scholarship by considering the role of psychosocial pathways underlying this association in a nationally representative sample in China. Specifically, using data from the latest 2021 wave of Chinese General Social Survey (CGSS), we examine the mediating effects of perceived work stress, work–family conflict and job satisfaction on the relation between nonstandard work schedules and employees’ self-rated mental health.

After controlling for self-rated physical health, overtime work, changing jobs during COVID-19 and demographics, baseline analyses revealed that nonstandard work schedules had a negative relationship with employees’ self-rated mental health, thereby Hypothesis 1 was supported. This finding aligns with previous research that reported significant associations between nonstandard work schedules and mental health (Cho, 2018; Frazier, 2023; Haines et al., 2008; Han, 2023; Lee et al., 2016; Niedhammer et al., 2022; Zhao et al., 2021a).

Furthermore, supporting Hypotheses 2, 3 and 4, we found that working nonstandard schedules was associated with increases in perceived work stress and work–family conflict as well as decreases in perceived job satisfaction, which in turn influenced the decrease in the self-reported score of mental health. Specifically, the independent mediation analysis revealed that perceived work stress, perceived work–family conflict and perceived job satisfaction accounted for about 17.3, 22.4 and 16.5%, respectively, of the total effect of the association between nonstandard work schedules and employees’ self-rated mental health, and the simultaneous mediation analysis revealed that the joint effect of all three mediators mediated about 36.2% of the total effect of the association between nonstandard work schedules and employees’ self-rated mental health, for work–family conflict, work stress and job satisfaction were correlated (Viegas and Henriques, 2021). Previous research has found that work satisfaction, work stress, and ability to control the work schedule were key contributing factors related to mental health via multiple linear regression analyses (Pitanupong et al., 2024), and we forward recognized that perceived work stress and job satisfaction mediated the association between work schedule and mental health. Our study is the first, to our knowledge, to explicitly test and confirm the mediating role of perceived work stress and job satisfaction in the association between work schedule and mental health.

On the other hand, work–family conflict mechanism has been proposed to account for the association between nonstandard work schedules and mental health in the USA adult samples (Cho, 2018), Australia employed parent samples (Zhao et al., 2021a), and Canada adult samples (Haines et al., 2008). Zhao et al.’s (2021a) study highlight the complex interplay between parents’ work schedules, work–family conflict and psychological distress. Cho’s (2018) study indicated a full mediation effect of work–family conflict, whereas Haines et al.’s (2008) study indicated a partial mediation effect of work–family conflict in the relationship between nonstandard work schedules and mental health. In our study, perceived work–family conflict was found to partially mediate the association between nonstandard work schedule and mental health, meanwhile, perceived work stress mechanism and perceived job satisfaction mechanism were also found in the multiple mediation model at one time, and these findings stand by Haines et al.’s (2008) study. Furthermore, Haines et al.’s (2008) research found that about 30% of the effect of shiftwork on depression was indirect through work–family conflict, and our independent mediation analysis indicated that about 22.4% of the effect of the association between nonstandard work schedule and self-rated mental health was indirect through perceived work–family conflict, but our simultaneous mediation analysis indicated that by adding perceived work stress and job satisfaction as mediators the proportion of the joint indirect effect raising up to about 36.2%. Moreover, our finding that relative to perceived work–family conflict, either perceived work stress or perceived job satisfaction mediates almost the same proportion of the relationship between nonstandard work schedules and employee’s self-rated mental health in the simultaneous mediation model is noteworthy, and we recommend that research examine the role of other psychosocial mechanisms for the association between nonstandard work schedules and mental health.

The results of this study provide for a more ‘precise’ understanding of the relationship between work schedule and mental health (Totterdell, 2005). We found that nonstandard work schedules cause increasing work stress and work–family conflict as well as decreasing job satisfaction, which can impact employees’ mental health. By including work–family conflict as a mediating variable, previous studies elucidated a social pathway between nonstandard work schedules and mental health besides biological pathway (Cho, 2018; Haines et al., 2008; Zhao et al., 2021a). Possibly one of the theoretical implications of this study is that by adding perceived job satisfaction and work stress as mediating variables, we extend social pathway to psychosocial pathway. Furthermore, our findings reveal that perceived work stress, perceived job satisfaction, and perceived work–family conflict mediate almost the same proportion of the total effect between nonstandard work schedules and employee self-rated mental health. In addition, the majority of research on work schedules takes place in a few countries (Totterdell, 2005), there is a need for more evidence-based research from China. To the best of our knowledge, this is the first nationally representative study in China to investigate work schedules.

### 6.1 Practical implications

The results of the study may also provide practical implications for employers and policy makers to make policies and interventions on employees’ mental health and occupational health. In light of the effects of psychosocial mechanisms, the mediating effects of perceived work stress, work–family conflict and job satisfaction provide new insights to improve the mental health of employees with nonstandard work schedules, not only by improving sleep quality, but also by reducing both perceived work stress and work–family conflict, as well as raising perceived job satisfaction. Employees themselves should also be wary of nonstandard work schedules that could undermine their mental health not only through reduce sleep quality but also through increase work stress and work–family conflict as well as decrease job satisfaction.

### 6.2 Limitations and future research

There are various study limitations to note. First, although CGSS had included 12 waves so far, we were only able to use one wave of data given the availability of the work schedules measure only in the “quality of work topical module” added in the latest 2021 wave. As such, we cannot speak to causality between nonstandard work schedules and mental health. We are aware that mental health may affect job satisfaction, work-family balance and work stress, therefore, there is potential for a bidirectional relationship between the factors considered in our study. Given the availability of data, future research could employ a longitudinal analysis to better understand the causal relationship of the mediating roles of work stress, work–family conflict and job satisfaction between work schedule and mental health. Second, our nonstandard work schedules measure is mainly a simple dichotomous measure. Although we divided and considered different types of nonstandard work schedules in sensitivity analysis, and achieved some findings on the rotating shift and irregular schedule employees, small sample size for fixed night shift (about 1.29%) prohibited any meaningful analysis on fixed night shift employees. Also, we did not account for dose–response relationships, such as the frequency of working nonstandard schedules. We would like to encourage future researchers to replicate our study using alternative data and measurement sources. Third but not last, this study may be limited by potential self-report bias for the variables in this study were measured using self-report instruments, and the self-rated mental health, perceived work stress, perceived work–family conflict and perceived job satisfaction measure used in our study are single-item, and future research should consider replicating the current findings with more standardized, objective and multidimensional measure tools.

Limitations notwithstanding, our study provides a novel contribution to the body of literature on the relationship between nonstandard work schedules and mental health by considering the mediating roles of perceived work stress, work family conflict and job satisfaction. Our findings reveal that psychosocial factors, not only work–family conflict, but also perceived work stress and job satisfaction, may play mediating roles on the association between nonstandard work schedules and mental health. Future research should examine other potential psychosocial mechanisms underlying the association between work schedule and mental health to establish why work schedule variable is associated with mental health outcome, and it is important for the advancement of knowledge (Haines et al., 2008). For example, organizational participation, social contact, and solitary activities are some of the problems which the shift worker may encounter with greater frequency than the day worker (Dunham, 1977), and these factors may play roles with mental health. Another example is that workers with nonstandard work schedules may have unequal work assignments perception (Kida and Takemura, 2024), and the organizational justice may be another potential psychosocial mechanism underlying the association between nonstandard work schedules and mental health. There is still much to know, and more research is needed to enhance our understanding of the relationships between work schedule and mental health.

## Data Availability

Publicly available datasets were analyzed in this study. This data can be found here: http://www.cnsda.org/index.php?r=projects/view&id=65635422.
